# Glycans Flanking the Hypervariable Connecting Peptide between the A and B Strands of the V1/V2 Domain of HIV-1 gp120 Confer Resistance to Antibodies That Neutralize CRF01_AE Viruses

**DOI:** 10.1371/journal.pone.0119608

**Published:** 2015-03-20

**Authors:** Sara M. O’Rourke, Ruengpung Sutthent, Pham Phung, Kathryn A. Mesa, Normand L. Frigon, Briana To, Navin Horthongkham, Kay Limoli, Terri Wrin, Phillip W. Berman

**Affiliations:** 1 Department of Biomolecular Engineering, Baskin School of Engineering, University of California Santa Cruz, Santa Cruz, California, United States of America; 2 National HIV Repository and Bioinformatics Center (Thailand), Department of Microbiology, Faculty of Medicine, Siriraj Hospital, Mahidol University, Bangkok, Thailand; 3 Monogram Biosciences, South San Francisco, California, United States of America; 4 Gladstone Institute of Virology & Immunology, San Francisco, California, United States of America; University of Melbourne, AUSTRALIA

## Abstract

Understanding the molecular determinants of sensitivity and resistance to neutralizing antibodies is critical for the development of vaccines designed to prevent HIV infection. In this study, we used a genetic approach to characterize naturally occurring polymorphisms in the HIV envelope protein that conferred neutralization sensitivity or resistance. Libraries of closely related envelope genes, derived from virus quasi-species, were constructed from individuals infected with CRF01_AE viruses. The libraries were screened with plasma containing broadly neutralizing antibodies, and neutralization sensitive and resistant variants were selected for sequence analysis. *In vitro* mutagenesis allowed us to identify single amino acid changes in three individuals that conferred resistance to neutralization by these antibodies. All three mutations created N-linked glycosylation sites (two at N136 and one at N149) proximal to the hypervariable connecting peptide between the C-terminus of the A strand and the N-terminus of the B strand in the four-stranded V1/V2 domain β-sheet structure. Although N136 has previously been implicated in the binding of broadly neutralizing monoclonal antibodies, this glycosylation site appears to inhibit the binding of neutralizing antibodies in plasma from HIV-1 infected subjects. Previous studies have reported that the length of the V1/V2 domain in transmitted founder viruses is shorter and possesses fewer glycosylation sites compared to viruses isolated from chronic infections. Our results suggest that vaccine immunogens based on recombinant envelope proteins from clade CRF01_AE viruses might be improved by inclusion of envelope proteins that lack these glycosylation sites. This strategy might improve the efficacy of the vaccines used in the partially successful RV144 HIV vaccine trial, where the two CRF01_AE immunogens (derived from the A244 and TH023 isolates) both possessed glycosylation sites at N136 and N149.

## Introduction

A major goal in HIV-1 research is the development of vaccines able to elicit protective broadly neutralizing antibodies (bNAbs). For many years, it was uncertain whether it was biologically possible for the human immune system to produce antibodies capable of neutralizing diverse isolates from genetically distinct clades of virus. However, over the last five years, a number of potent broadly neutralizing monoclonal antibodies (bN-MAbs) have been isolated from rare HIV-1-infected individuals, termed elite neutralizers, or ENs [1–6]. The discovery that ENs are observed among people from different parts of the world, infected with viruses from different clades, suggests that the ability of humans to make bNAbs is more common than previously suspected. These results provide hope that an effective HIV vaccine may be possible, regardless of the genetic background of the host or the virus. However, the ability of humans to make bNAbs is counterbalanced by the ability of HIV-1 to evade antibody-mediated neutralization [7,8]. It is likely that the development of effective vaccines and therapeutic antibodies against HIV will depend on understanding the mechanisms of neutralization resistance, as was the case with the development of effective anti-retroviral drugs [9–12].

In previous studies [13–15], we described a genetic approach, termed swarm analysis, to study the problem of neutralization resistance. This method makes use of the closely related swarm of virus quasi-species that evolve in each HIV-1-infected individual. The members of the swarm represent naturally occurring, and biologically relevant, isoforms that allow us to study the specificity of neutralizing antibodies in plasma and the specific mutations that facilitate immune escape. In this paper, we have studied virus quasi-species present in plasma from a cohort of injection drug users (IDUs) in Thailand infected with CRF01_AE viruses [16]. We have recovered viral sequences from these specimens and have used them to identify naturally occurring polymorphisms that confer resistance or sensitivity to neutralization by polyclonal and monoclonal antibodies from ENs. Structural data for trimeric gp140 [17–19] and bN-MAbs to glycan-dependent epitopes (GDEs) [20–24] allows us to study the antigenic structure of the V1/V2 and V3 domains, and the role of carbohydrates in determining the sensitivity and resistance to antibody-mediated neutralization. The results may inform the selection of immunogens to be included in candidate HIV vaccines.

## Materials and Methods

### Plasma, monoclonal antibodies, and entry inhibitors

Cryopreserved plasma specimens from injection drug users (IDUs) who became infected with HIV-1 during the course of the VAX003 Phase 3 clinical trial [25] were obtained from Global Solutions for Infectious Diseases (GSID, South San Francisco, CA). All of the specimens used in this study were obtained within six months of initial infection. For neutralization assays, plasma from individuals infected with either CRF01_AE viruses from Thailand or clade B isolates from North America were used as the source of bNAbs. Thai plasma possessing bNAbs to HIV-1 were recovered from rejected blood donations, and initially characterized by Dr. Ruengpung Sutthent (Siriraj Hospital, Bangkok, Thailand). After screening multiple samples using a TZM-bl neutralization assay [26], three plasma (T500105, T500107, and T500208) were selected for use in these studies. Plasma Z23, from a clade B-infected subject possessing bNAbs, was provided by Monogram Biosciences, Inc. (South San Francisco, CA) and used as a positive control and comparator in these studies. The bN-MAbs b12 [27], 4E10 [28], PG9 and PG16 [23] were obtained from Polymun A.G. (Vienna, Austria). VRC01 [29] was obtained from the NIH AIDS Reagent Repository. PGT121, PGT122, and PGT128 were a generous gift from Dennis Burton of The Scripps Research Institute, La Jolla, CA. The antiviral compound CD4-IgG has been described previously [30,31] and was provided by GSID. The antiviral drug enfuvirtide (Fuzeon) was purchased from Roche, Inc. (Basel, Switzerland).

### Construction of envelope gene libraries and pseudoviruses

Libraries of envelope (*env*) glycoprotein genes for swarm analysis were recovered by Monogram Biosciences (South San Francisco, CA) using a modification of a commercially available clinical assay (PhenoSense HIV Entry Assay) developed to detect antiviral drug resistance. Briefly, cryopreserved plasma at a single time point was amplified by RT-PCR and the resulting mixture of *env* genes was cloned into an expression vector as described [32–34] and widely used [2,7,13–15,23,24,35,36]. For this purpose, libraries of *env* clones were prepared from 36 plasma specimens. From each library, 12–24 clones were isolated and tested for infectivity, and at least 10 clones with robust infectivity were chosen for subsequent neutralization studies.

### Sequencing, mutagenesis, and bioinformatic analysis

Plasmids containing cloned Env glycoproteins were sequenced using Sanger sequencing at either Monogram Biosciences or the University of California Sequencing Facility (Berkeley, CA). The sequences of the three pairs of neutralization-sensitive and -resistant Env genes described in this study have been deposited in GenBank (accession numbers JX848346-JX848351). Mutations were introduced into HIV-1 *env* genes by sequential point-by-point mutagenesis as described previously [13–15] using the QuikChange Lightning kit (Agilent, Santa Clara, CA), followed by confirmatory sequencing. Sequence alignment was performed within Jalview [37] using MAFFT alignment [38] with default parameters. Because of insertions of variable length, the alignment of the hypervariable sequence between positions 132 and 150 in the V1 domain was adjusted manually to maximize the homology. Sequence numbering is provided with reference to the HXB2 reference sequence (GenBank sequence AF033819). Molecular modeling utilized PyMOL [39]. Lengths for the V1 domain were calculated as beginning at C131 and ending at C157. V2 lengths were calculated as beginning at 158 and ending at position 196. Predicted N-linked glycosylation sites (PNGS) were identified using the consensus motifs NXS/T. For the V1 domain PNGS counts included positions from 130–156 and V2 PNGS counts included positions 157–197.

### Virus neutralization and receptor tropism assays

Virus neutralization assays were carried out at Monogram Biosciences using the same system as used by many investigators ([2,7,13–15,23,24,33]. Briefly, this assay involved pre-incubation of pseudovirions with inhibitors of infection (antibodies or fusion inhibitors) followed by transfer of the mixture to the U87 indicator cell line co-expressing CD4 and chemokine receptors, as described in detail elsewhere [7,32]. Neutralization data reported represent IC_50_ values calculated from plasma dilution curves. For initial screening, we used a starting dilution of 1:40 for the Thai plasma, and a 1:100 dilution of the Z23 plasma. In all other experiments, we used a starting plasma dilution of 1:100. The neutralization assays carried out at Monogram Bioscience were performed according to Good Laboratory Practices and using protocols approved under Clinical Laboratory Improvements Amendment (CLIA). Each assay included acceptability criteria to ensure that inter-assay variation between IC_50_s, measured with reference standards, fell within 2.5-fold 95% of the time. The co-receptor usage for each *env* gene was determined with the Trofile assay developed at Monogram Biosciences [36].

## Results

In these studies, we sampled the swarm of viruses that occur in each HIV-infected individual to characterize polymorphisms that determine neutralization sensitivity and resistance in CRF01_AE viruses found in Thailand. We made use of a panel of four plasma from HIV+ individuals known to possess antibodies able to neutralize CRF01_AE viruses. This panel included three plasma from unrelated individuals infected with CRF01_AE viruses (T500105, T500107, and T500208) and one plasma, Z23, from a clade B-infected EN that has been used as a positive control in previous neutralization studies by multiple investigators [2,7,13–15,32]. None of the plasma were obtained from donors receiving anti-viral therapy and no inhibitory activity was detected against the retroviral aMLV control virus. We found (Table 1) that two of the plasma, T500105 and T500208, neutralized Tier 1 clade B isolates and most primary CRF01_AE Thai isolates to varying degrees. However, one plasma, T500107, exhibited the extremely broad cross clade neutralizing activity characteristic of EN plasma [40]. In many cases, the neutralization titers obtained with the T500107 plasma were an order of magnitude higher than those observed with the well-characterized Z23 plasma.

**Table 1:** Neutralization of diverse HIV-1 isolates by polyclonal antibodies in plasma of HIV-1-infected subjects from Thailand and North America.

		Neutralization antibody titer (IC_50_) for indicated human HIV+ plasma sample^a^			
**Virus**	**Clade**	**T500105**	**T500107**	**T500208**	**Z23**
**SF162**	B	**3023**	**17785**	**532**	**17611**
**MN**	B	**4145**	**3256**	**1370**	**9417**
**TRO**	B	<40	**1844**	<40	**266**
**JRFL**	B	<40	**687**	<40	**431**
**BG1168**	B	<40	<40	<40	**120**
**QHO692**	B	<40	**147**	<40	<100
**REJO**	B	<40	**565**	<40	**306**
**APV-16**	B	<40	**1142**	<40	**569**
**92BR020**	B	<40	**1125**	<40	**224**
**PVO**	B	<40	**568**	<40	**403**
**MGRM-A-002**	A	<40	**1795**	<40	**120**
**MGRM-A-006**	A	<40	**2044**	<40	**166**
**94UG103**	A	**44**	**405**	<40	**111**
**93IN905**	C	**107**	**2297**	**69**	**217**
**MGRM-C-003**	C	41	<40	<40	<100
**MGRM-D-006**	D	<40	**142**	<40	**160**
**MGRM-D-009**	D	**70**	<40	<40	**243**
**92TH021**	CRF01_AE	**56**	**3315**	**419**	**325**
**107751_053**	CRF01_AE	**51**	**3066**	**125**	**612**
**107726_013**	CRF01_AE	**119**	**1593**	**164**	**369**
**107724_036**	CRF01_AE	**136**	**2621**	**183**	**135**
**107732_186**	CRF01_AE	<40	**751**	**3487**	**223**
**107747_048**	CRF01_AE	**80**	**5734**	**439**	**168**
**113039_158**	CRF01_AE	**182**	**469**	**98**	**609**
**142900_061**	CRF01_AE	**126**	**2393**	<40	**217**
**JRCSF**	B	<40	**2585**	<40	**282**
**NL4–3**	B	<40	**74**	<40	**1734**
**aMLV**		<40	<40	<40	<100

^a^The neutralizing titer (IC_50_) is defined as the reciprocal of the plasma dilution that produced a 50% inhibition in relative light units (RLU) in U87 target cells infected with pseudoviruses expressing a luciferase indicator gene under transcriptional control of the HIV-1 Tat gene. Values in bold represent significant neutralization titers that are at least three times greater than those observed against the negative control (aMLV).

These plasma were then used as described previously [13–15] to screen libraries of pseudoviruses constructed from the closely related *env* gene quasi-species found in the plasma of HIV-1 infected individuals. Overall we measured neutralization sensitivity and resistance in *en*v gene libraries from 36 individuals likely to have become infected with HIV-1 through injection drug use during the course of the VAX003 vaccine trial in Thailand [25]. Because the AIDSVAX B/E vaccine was ineffective in preventing new infections in this trial, and because phylogenetic analysis of the sequences of viruses from the vaccine and placebo groups failed to show clustering based on treatment [16], the samples analyzed were selected at random without regard for treatment group assignments. At least 10 independent *env* gene clones from each individual were screened for neutralization sensitivity with the panel of plasma described above. We found that most *env*s were sensitive to neutralization by T500107, but some were more sensitive than others. In contrast, both neutralization-sensitive and -resistant viruses were observed with the T500105 and T500208 plasma. Based on initial studies (S1–S3 Tables), we selected libraries of viruses from three subjects (107747, 113035, and 142902) for further study based on two criteria: 1) the identification of pairs of CCR5-dependent *env* clones that exhibited a reproducible phenotypic difference in neutralization sensitivity to one or more of the Thai plasma; and 2) Envs with fewer than 20 amino acid differences per pair of neutralization sensitive/resistant variants.

### Neutralization sensitivity/resistance in viruses from subject 107747

**Fig 1:**
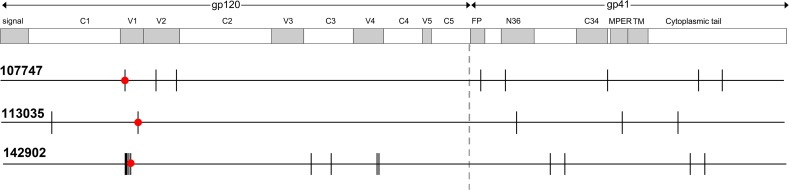
Diagram of sequence differences between pairs of neutralization-sensitive and -resistant Envs of CRF01_AE viruses. The locations of sequence differences between wildtype neutralization-resistant (wtR) and wildtype neutralization-sensitive (wtS) envelope genes from subjects 107747, 113035, and 142902 are indicated by vertical lines. The location of predicted N-linked glycosylation sites in the V1 domain that alter neutralization sensitivity is marked by red circles.

**Fig 2:**
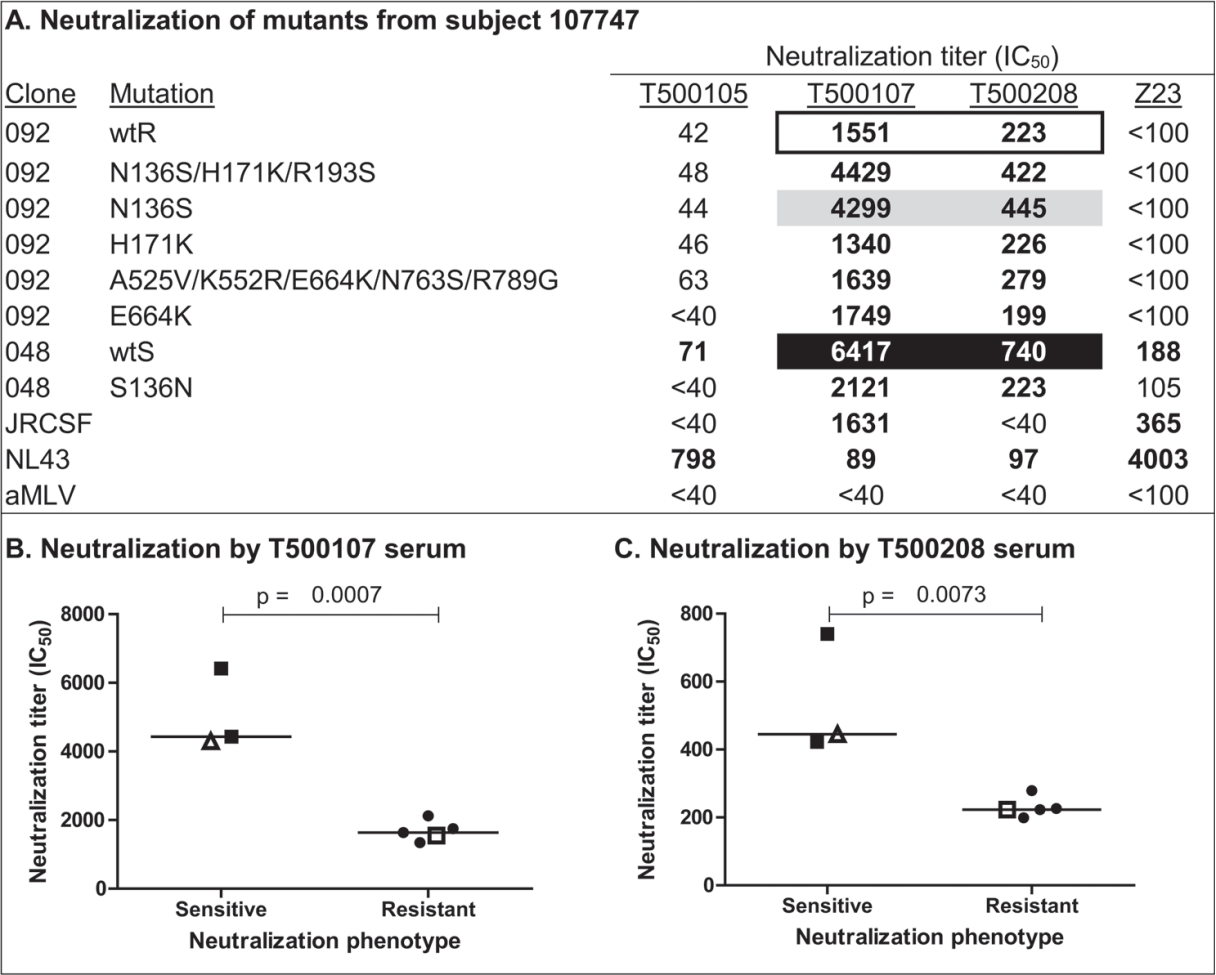
Mutational analysis to map residues responsible for differences in sensitivity and resistance in Envs from subject 107747. Amino acids from the neutralization-sensitive clone (048) were systematically inserted into the neutralization-resistant Env (092). (A) Effect of sequence polymorphisms on neutralization by plasma from four HIV-1 infected subjects. The neutralizing antibody titer (IC_50_) is defined as the reciprocal of the plasma dilution that produces a 50% inhibition in target cell infection. Values in bold represent significant neutralization titers that are at least three times greater than those observed against the negative control (aMLV). Panel A, open rectangle, indicates neutralization titers for the wildtype resistant (wtR) clone; black rectangle, indicates neutralization titers for the wildtype sensitive (wtS) clone; gray rectangle, indicates the single amino acid substitution that converted the neutralization-resistant Env into a neutralization-sensitive Env. Panels B and C represent graphs and statistical analysis of neutralization-sensitive and -resistant viruses with the T500107 and T500208 plasma, respectively. Panels B and C, closed square (■) indicates neutralization titers of wtS clone 048; open square (□) indicates neutralization titer of wtR clone 092. Open triangles (△) indicate neutralization titers of wtR clone 092 incorporating the N136S mutation. Closed circles (●) indicate neutralization by the other mutants listed in panel A. Statistical significance was calculated using an unpaired t test (GraphPad Prism).

The first pair of viruses analyzed was from subject 107747. We found that clone 048 was approximately five-fold more sensitive to neutralization by the T500107 plasma than clone 092, and three-fold more sensitive to the T500208 plasma than clone 092 (S1 Table). Clones 048 and 092 were designated the wildtype sensitive (wtS) and wildtype resistant (wtR) Envs respectively. Alignment of sequences showed that the eight amino acid differences between clones 092 and 048 were located in the V1, V2, and gp41 regions (Fig. 1). Next we carried out stepwise amino acid replacement experiments, where amino acids in the wtR clone 092 were replaced with the corresponding amino acids of wtS clone 048. The results of these studies are shown in Fig. 2. These studies showed that replacement of asparagine (N) at position 136 with serine (S) in the V1 domain was able to confer the neutralization-sensitive phenotype to clone 092 when measured with both the T500107 and T500208 plasma (Fig. 2A-2C). Conversely, replacement of S at 136 with N in the wtS clone 048 increased neutralization resistance with both plasma. Examination of the sequences flanking position 136 showed that the N136S mutation resulted in the loss of a predicted N-linked glycosylation site (PNGS) with the Asn-X-Ser/Thr motif. Thus neutralization resistance was observed when the PNGS was present, and neutralization sensitivity was observed when the PNGS was absent. Mutation of polymorphic residues in the V2 domain and gp41 had no effect on neutralization sensitivity and resistance. Accordingly, the two unrelated plasma (T500107 and T500208) both appeared to possess a population of neutralizing antibodies that was inhibited by glycosylation at position 136. The 136N polymorphism observed in clone 092 resulted from a single G-A nucleotide substitution. Mutations of this type commonly occur during reverse transcription [41–43] and have long been recognized as a strategy used by HIV-1 for immune escape [44,45].

### Neutralization sensitivity/resistance in viruses from subject 113035

**Fig 3:**
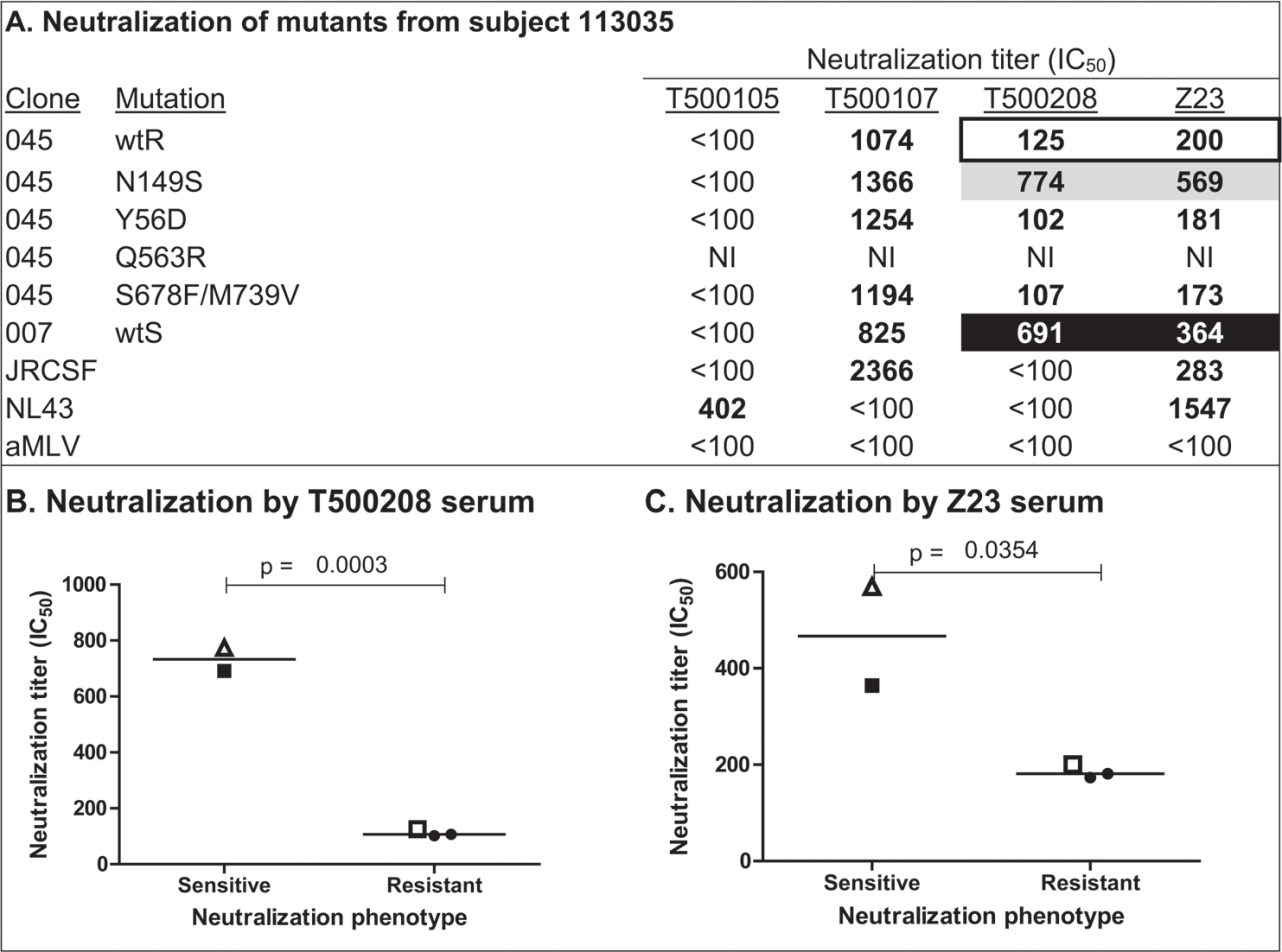
Mutational analysis to map residues responsible for differences in neutralization sensitivity and resistance in Envs from subject 113035. Amino acids from the neutralization-sensitive clone (007) were systematically inserted into the neutralization-resistant Env (045). (A) Effect of amino acid polymorphisms on neutralization by plasma from four HIV-1 infected subjects. The neutralizing antibody titer (IC_50_) is defined as the reciprocal of the plasma dilution that produces a 50% inhibition in target cell infection. Values in bold represent significant neutralization titers that are at least three times greater than those observed against the negative control (aMLV). Panel A, open rectangle, indicates neutralization titers for the wildtype resistant (wtR) clone; black rectangle, indicates neutralization titers for the wildtype sensitive (wtS) clone; gray rectangle, indicates the single amino acid substitution that converted the neutralization-resistant Env into a neutralization-sensitive Env. NI, indicates a non-infectious mutant. Panels B and C represent graphs and statistical analysis of neutralization of 113035 mutants with the T500208 and Z23 plasma, respectively. Panels B and C, closed square (■) indicates neutralization titers of wtS clone 007; open square (□) indicates neutralization titer of wtR clone 045. Open triangles (△) indicate neutralization titers of wtR clone 045 incorporating the N150S mutation. Closed circles (●) indicate neutralization by the other mutants listed in panel A. Statistical significance was calculated using an unpaired t test.

We next examined the Envs from subject 113035 for differences in neutralization sensitivity and resistance (S2 Table). We found differences in neutralization sensitivity and resistance to both the T500107 and T500208 plasma. Comparison of the sequence of the neutralization-resistant clone 045 with the more neutralization-sensitive clone 007 revealed five amino acid differences. These were located in the C1 and V1 domains of gp120 and in the N36 helix, the MPER, and cytoplasmic tail of gp41 (Fig. 1). Stepwise mutagenesis (Fig. 3A) to replace amino acids from the wtS clone into the wtR clone sequence eliminated residues in the C1 or gp41 domains as being responsible for the difference in neutralization sensitivity. The Q563R mutation in the N36 helix of gp41 from subject 113035 resulted in an Env with low infectivity that could not be assayed. However, the replacement of asparagine (N) with serine (S) at position 149 (N149S) in the V1 domain was sufficient to increase T500208-mediated neutralization five-fold (Fig. 3A-3C). Additionally, the N149S mutation increased the sensitivity to neutralization by the clade B Z23 plasma. The presence of a glycosylation site at position 149 appears to block the binding of neutralizing antibodies in these subjects infected with either CRF01_AE or clade B isolates. Examination of the sequences flanking this position revealed that the N149S mutation destroyed a PNGS and enhanced neutralization sensitivity in a manner similar to the effect of the N136S mutation described for 107747. As with 107747_092_N136S, this 113035_045_N149S polymorphism resulted from a single G-A substitution.

### Neutralization sensitivity/resistance in viruses from subject 142902

**Fig 4:**
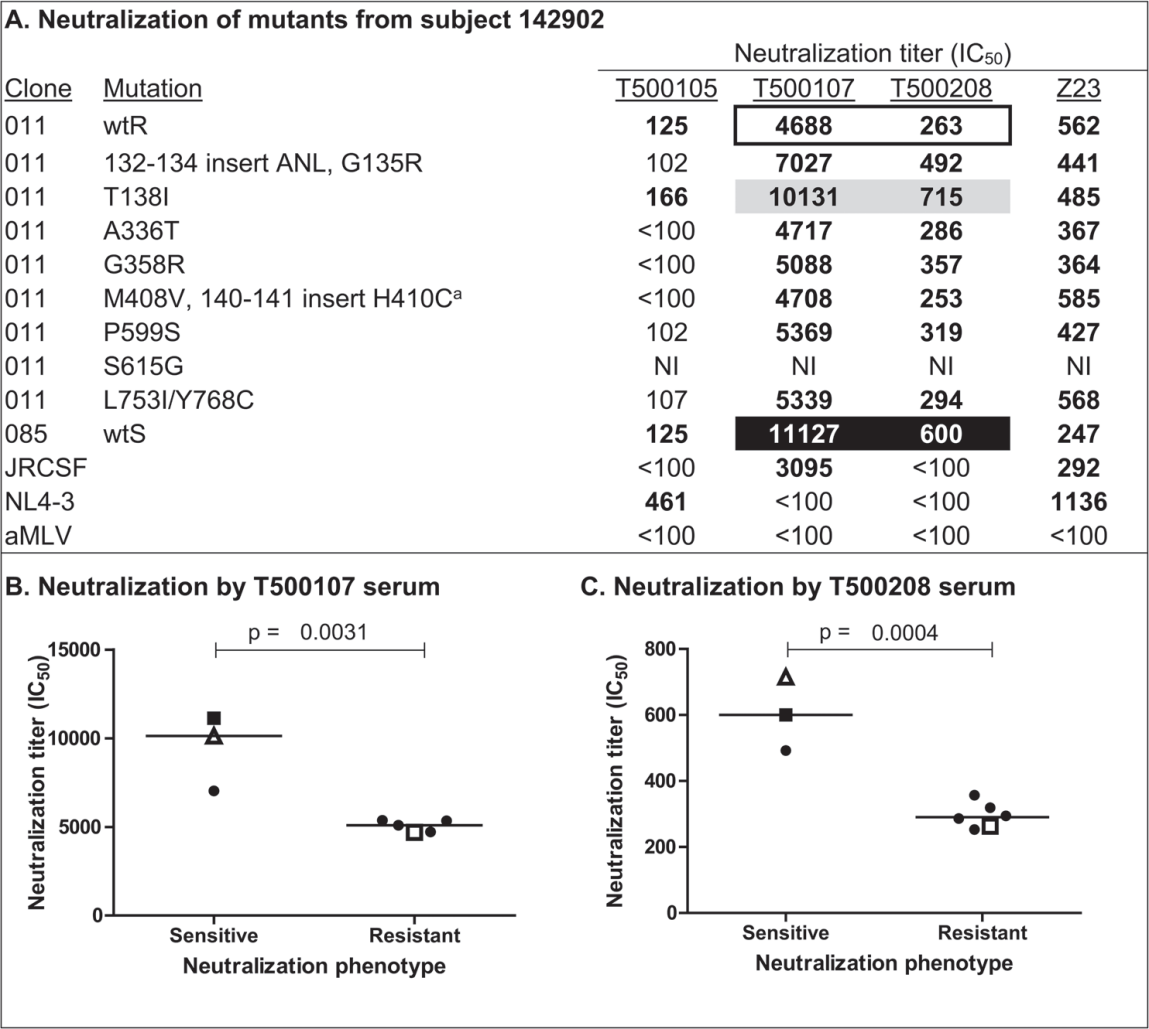
Mutational analysis to map residues responsible for differences in neutralization sensitivity and resistance in Envs from subject 142902. Amino acids from the neutralization-sensitive clone (085) were systematically inserted into the neutralization-resistant Env (011). (A) Effect of amino acid polymorphisms on neutralization by plasma from four HIV-1 infected subjects. The neutralizing antibody titer (IC_50_) is defined as the reciprocal of the plasma dilution that produces a 50% inhibition in target cell infection. Values in bold represent significant neutralization titers that are at least three times greater than those observed against the negative control (aMLV). Panel A, open rectangle, indicates neutralization titers for the wildtype resistant (wtR) clone; black rectangle, indicates neutralization titers for the wildtype sensitive (wtS) clone; gray rectangle, indicates the single amino acid substitution that converted the neutralization-resistant Env into a neutralization-sensitive Env. NI, indicates a non-infectious mutant. Panels B and C represent graphs and statistical analysis of neutralization-sensitive and -resistant viruses with the T500107 and T500208 plasma, respectively. Panels B and C, closed square (■) indicates neutralization titers of wtS clone 085; open square (□) indicates neutralization titer of wtR clone 011. Open triangles (△) indicate neutralization titers of wtR clone 011 incorporating the T137I mutation. Closed circles (●) indicate neutralization by the other mutants listed in panel A. Statistical significance was calculated using an unpaired t test.

Viruses from subject 142902 were then assayed for sensitivity to neutralization (S3 Table). We found that the viruses from this subject were unusual in that they were all sensitive to neutralization by all four HIV+ plasma and extremely sensitive to neutralization by the T500107 plasma (Fig. 4). However, the pattern of neutralization sensitivity and resistance was similar for both the T500107 and T500208 plasma. Comparison of the sequences of the wtR clone 011 and wtS clone 085 revealed that there were 13 amino acid differences between the wtS and wtR variants. These included five polymorphisms in the V1 domain (one of which was a three amino acid insertion), two amino acid differences in the C3 domain; two amino acid differences in the V4 domain; and four substitutions in gp41 (Fig. 1). Systematic replacement of amino acids from the wtS 085 clone into the wtR 011 backbone (Fig. 4A) showed that a single mutation of threonine (T) to isoleucine (I) at position 138 (T138I) destroyed the glycosylation site at position N136, and was able to rescue the neutralization-sensitive phenotype observed with both the T500107 and T500208 plasma (Fig. 4A-C). The other mutations in the V1 domain, that included a three-amino-acid insertion (132–134) and the replacement of glycine (G) with arginine (R) at position 135, appeared to have little or no effect on neutralization. A single S to G mutation at position 615 in the N36 helix (S615G) reduced infectivity below the threshold required for assay, and none of the other seven mutations altered neutralization sensitivity to the panel of four HIV+ plasma (Fig. 4A). The T138I mutation thus represented the third independent case where the disruption of a PNGS in the V1 domain increased neutralization sensitivity in a CRF01_AE virus.

### Effect of mutations in the V1 domain on neutralization by bN-MAbs and virus entry inhibitors

We next examined the effect of the mutations at PNGSs in the V1 domain on neutralization by a panel of potent neutralizing monoclonal antibodies (MAbs) and virus entry inhibitors (Table 2). When the three pairs of neutralization sensitive/resistant CRF01_AE viruses were examined, all were relatively resistant to CD4-IgG with IC_50_ values from 11 to >20μg/ml. This result is consistent with previous observations showing that clinical isolates are 1–2 logs more resistant to CD4-IgG than lab-adapted strains [46]. When we examined sensitivity to the VRC01 MAb, also targeting the CD4 binding site, we found that all of the viruses tested were sensitive to neutralization by this MAb regardless of whether the PNGSs in the V1 domain were present. When we examined the sensitivity of these clones to the b12 bN-MAb which targets residues near the CD4 binding site, we found all were resistant, confirming previous reports that CRF01_AE viruses are resistant to neutralization by this MAb [47,48]. Additional studies utilized the virus entry inhibitor enfuvirtide (Fuzeon) and the bN-MAb 4E10, both of which target sites in gp41 including the membrane proximal external region (MPER)[49]. We found that all of the viruses tested were sensitive to neutralization by these inhibitors.

**Table 2:** Effect of amino acid substitutions on neutralization by bN-MAbs and entry inhibitors^a^.

Subject	Clone	Mutation	b12	PG9	PG16	PGT121	PGT122	PGT128	VRC01	4E10	CD4-IgG	Fuzeon
**107747**												
	092	wtR	>20	**0.69**	>20	>25	>25	**0.0255**	**0.31**	**1.20**	**11.02**	**0.03**
	048	wtS	>20	**0.22**	**1.54**	>25	>25	**0.0135**	**0.20**	**1.35**	**14.25**	**0.03**
	092	N136/S	>20	**0.55**	>20	>25	>25	**0.0189**	**0.19**	**1.66**	**11.38**	**0.02**
**113035**												
	045	wtR	>20	**0.09**	**0.01**	>25	>25	>25	**0.13**	**0.36**	>20	**0.09**
	007	wtS	>20	**0.02**	**0.01**	>25	>25	**0.4709**	**0.10**	**0.07**	>20	**0.74**
	045	N149S	>20	**0.04**	**0.01**	>25	>25	**1.5450**	**0.07**	**0.15**	>20	**0.07**
**142902**												
	011	wtR	>20	**0.05**	**0.01**	>25	>25	**0.0304**	**0.11**	**1.65**	>20	**0.16**
	085	wtS	>20	**0.04**	**0.01**	>25	>25	**0.0178**	**0.08**	**1.36**	>20	**0.10**
	011	T138I	>20	**0.05**	**0.01**	>25	>25	**0.0091**	**0.11**	**2.11**	>20	**0.12**
**JRCSF**		control	**0.35**	**<0.01**	**<0.01**	**0.0358**	**0.0644**	**0.0081**	**0.34**	**11.07**	**6.34**	**0.14**
**NL4–3**		control	**0.23**	**2.37**	**0.08**	>25	>25	>25	**0.31**	**6.02**	**0.05**	**0.27**
**aMLV**		control	>20	>10	>10	>25	>25	>25	>10	>20	>20	>20

^a^Values in bold represent significant neutralization titers (IC_50_ μg/mL) that are at least three times greater than those observed against the negative control (aMLV).

**Fig 5:**
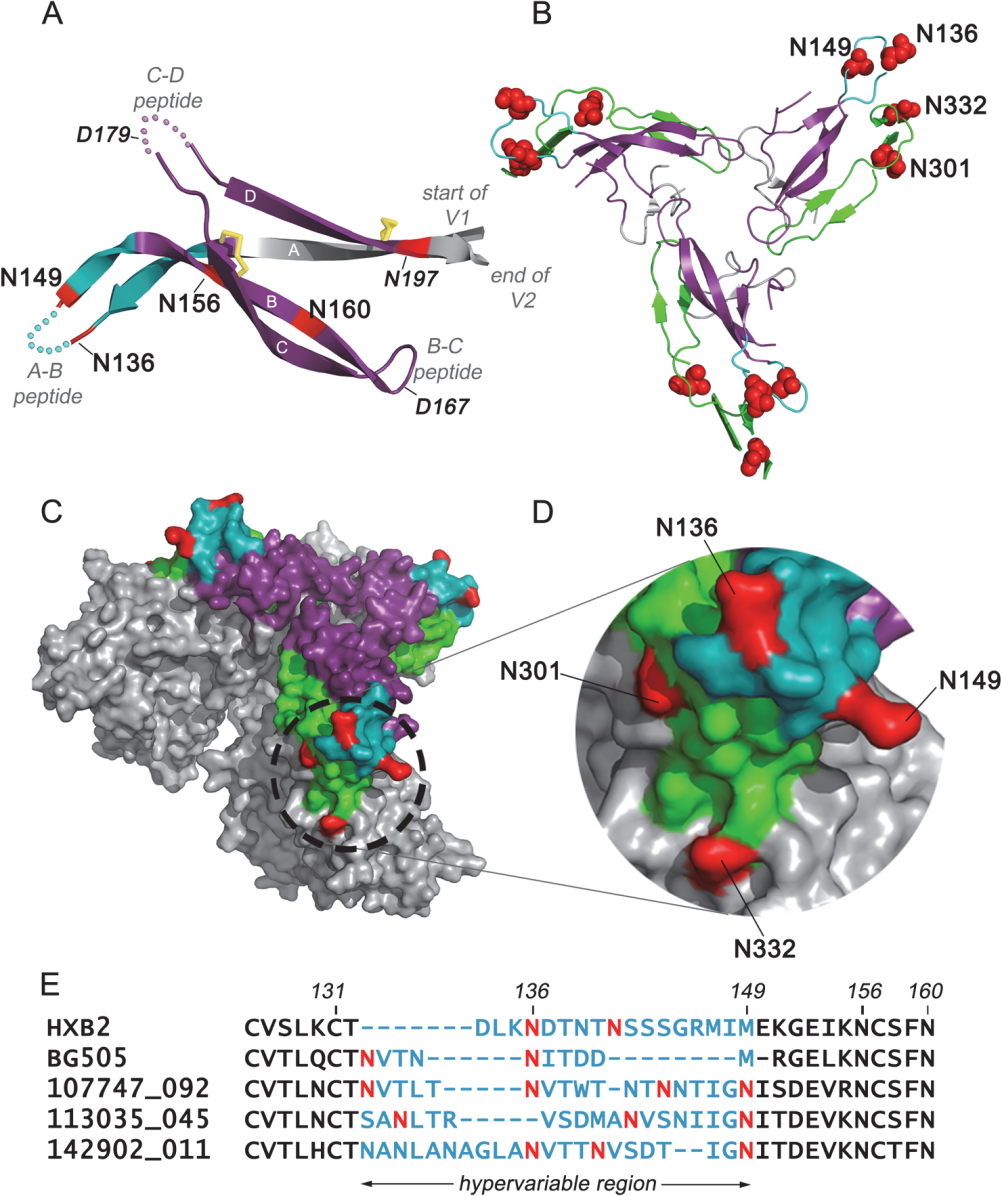
Location of mutations identified by swarm analysis that mediate resistance to neutralization in CRF01_AE viruses. (A) Ribbon diagram of the V1/V2 domain is based on the structure (PDB 3U4E) of McLellan et al. [20]. Strands A-D of the four-stranded β-sheet structure are indicated. Locations of predicted glycosylation sites and mutations that determine sensitivity and resistance to neutralizing polyclonal or monoclonal antibodies are indicated in red. Mutations at glycosylation sites in the V1 domain of CRF01_AE viruses that determine sensitivity and resistance to neutralization are located at the junction of the A and B strands at positions 136 and 149. Also shown are the relative locations of mutations at positions 167, 180, and 197 described previously [15], that conferred sensitivity and resistance to neutralization. The locations of glycosylation sites at positions 156 and 160 in the B strand required for the binding of the PG9 MAb [20,23] are indicated. Disulfide bonds are shown in yellow. (B) Diagram of trimeric gp140 derived from the PDB 4NCO structure of Julien et al. [18]. In this structure, sequences in the V1/V2 domain are colored violet and sequences from the V3 domain are colored green. Red balls indicate the location of the glycosylation sites at 136 and 149 identified by swarm analysis and the locations of the N301 and N332 glycosylation sites in the V3 domain required for the binding of the PGT121, PGT122, and PGT128 bN-MAbs. (C) Surface diagram of the gp140 trimer derived from the PDB 3J5M structure of Lyumkis et al. [17], shows the location of the glycans at N136, N149, N301, and N332 recognized by neutralizing monoclonal and polyclonal antibodies. In panels C and D, sequences from the V1/V2 domain are shaded violet and sequences from the V3 domain are shaded green. The hypervariable connecting peptide between the A and B strands is shaded cyan. (D) Magnified view of the surface of the gp140 trimer showing the locations of glycosylation sites (red) at N136, N149, N301, and N332 that affect the binding of bNAbs. (E) Alignment of sequences from the CRF01_AE viruses described in this paper along with the HXB2 reference sequence and the sequence of the BG505 Env used for determination of the 3-dimensional structure of the gp140 trimer [17,18]. The hypervariable region is indicated in cyan. The location of PNGS in the hypervariable domain is indicated in red. Sequence number is provided with reference to the HXB2 sequence and to the numbering of BG505 [18].

We then tested the sensitivity of the CRF01_AE viruses to neutralization by bN-MAbs targeting glycan-dependent epitopes (GDEs). When we examined the activity of the PG9 MAb that recognizes a GDE involving PNGS at positions 156 and 160 in the V1/V2 domain, we found all of the Envs were sensitive to neutralization by PG9 regardless of the presence or absence of PNGS in the V1 domain. This result was somewhat surprising, since the glycans recognized by PG9 (N156 and N160) occur in relatively close proximity to the V1 glycans proximal to the junction of the A and B strands [18,20] (Fig. 5A). Similarly, no difference in neutralization sensitivity was seen between wtS and wtR clones from subjects 113035 and 142902 using the PG16 MAb. Although a difference in sensitivity to PG16 was observed between clones 092 and 048 from 107747, this difference was clearly not the result of the N136S mutation and was likely the result of one of the other two mutations in the V2 domain. We then examined the sensitivity of these viruses to neutralization by the PGT128 bN-MAb. We found that inactivation of the N136 glycosylation site in the 107747 virus (by a N136S mutation) had no effect on neutralization by PGT128. However, inactivation of the same glycosylation site in the 142902 virus (by a T138I mutation) appeared to have a modest (three-fold) effect. In contrast, deletion of the glycosylation site at position 149 in the 113035 virus (N149S) resulted in a marked (>16-fold) increase in PGT128 neutralization sensitivity. This result suggested that glycosylation at N149 occludes the epitope recognized by PGT128. Thus, glycosylation at N149 near the N-terminus of the B strand in the four-stranded V1/V2 domain β-sheet structure is able to inhibit binding by PGT128, an antibody that recognizes a GDE (N301 and N332) in the stem of the V3 domain [18,21]. We next examined sensitivity to neutralization by PGT121 and PGT122 that are members of the PGT128 family. Neither of these antibodies was effective against any of the viruses tested. This result is likely due to the fact that CRF01_AE viruses typically lack the N332 glycosylation site often required for PGT121 and PGT122 binding. While it has been reported [22] that these antibodies can sometimes bind to envelopes from other clades where N332 is replaced by N334, this does not appear to be the case for the viruses we have studied or other CRF01_AE viruses.

### Sequence analysis of the V1 domain

**Fig 6:**
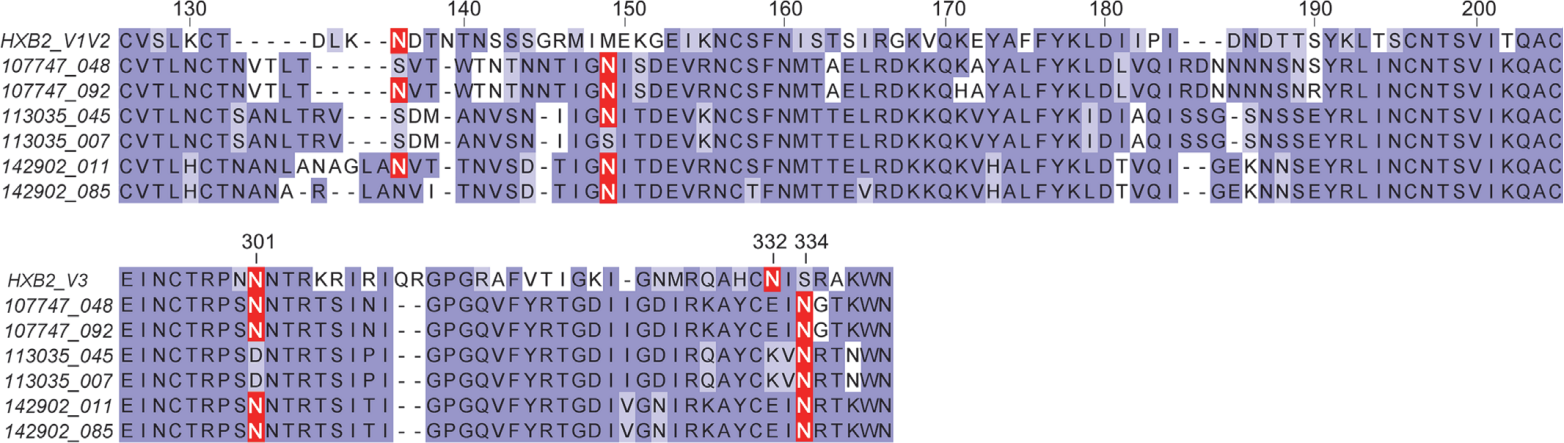
Alignment of sequences from the V1/V2 and V3 domains of gp120. The location of glycosylation sites important for the binding of monoclonal and polyclonal antibodies effective in neutralizing the viruses used in this study are indicated in red. Sequence numbering relative to the HXB2 reference standard is provided.

Based on the results obtained above, we wanted to characterize the sequence variation in the V1/V2 and V3 domains of the viruses analyzed in this study and in CRF01_AE viruses in general. An alignment of sequences from the V1/V2 and V3 domains of the viruses analyzed in this study is provided in Fig. 6. A comparison of the key features of the V1/V2 and V3 domains from these sequences is provided in Table 3. It can be seen that the length of the V1 domain varied from 27 to 32 amino acids. When we aligned and compared 563 sequences from 182 early CRF01_AE infections from the VAX003 vaccine trial using MAFFT [50], we found that the CRF01_AE viruses had a median V1 domain length of 29.3 amino acids in length and possessed 4.8 PNGS [51]. Our study showed a relatively high level of conservation of the PNGS at position 136 near the N-terminus of the hypervariable A-B connecting peptide, and at position 149 adjacent to the end (C-terminus) of this connecting peptide. Thus, 91.7% (516/563) of sequences possessed a PNGS at position 136 +/- 3, and 79.8% (449/563) sequences possessed a PNGS at position 149. We also examined the frequency of PNGS at positions 332 and 334 at the stem of the V3 domain (Table 3). We observed that PNGSs occurred at position 334 in 90.4% of CRF01_AE viruses (509/563), whereas a glycosylation site occurred at 332 in only 3.4% of viruses (19/563). Thus most CRF01_AE viruses lacked the N332 glycosylation site required for the binding of PGT121- and PGT122-like MAbs [22].

**Table 3:** Length and number of PNGS in the V1/V2 domain of gp120 from CRF01_AE viruses.

	V1		V2		V1+V2	
	Length	PNGS^1^	Length	PNGS^2^	Length	PNGS^1,2^
**107747_048**	27	5	42	3	69	8
**107747_092**	28	6	42	3	70	9
**113035_045**	29	5	41	3	70	8
**113035_007**	29	4	41	3	70	7
**142902_011**	32	5	40	4	72	9
**142902_085**	29	3	40	3	69	6
* **VAX003**	29.3	4.84	40.88	3.15	70.14	7.99

*VAX003 values represent mean titers obtained from the analysis of 563 sequences from 182 individuals. The amino acids in the V1 and V2 domains were based on the structure of Leonard et al. [52] with numbering based on the HXB2 reference strain. The V1 domain corresponds to positions 131–156 and the V2 domain corresponds to positions 157–196. ^1^ includes N130; ^2^ includes N197.

### Structural interpretation of antibody binding and neutralization results

To better understand the relationship between envelope structure and neutralization resistance, we plotted the approximate location of the positions of N136 and N149 onto 3-dimensional structures of envelope proteins that have been solved [17,18,20] (Fig. 5A-5D). Although the sequences in the hypervariable region between the A and B strands of the proteins crystallized to date are shorter than the V1 and V2 domains of the CRF01_AE viruses described in our study (Fig. 5E), the locations of the C-terminus of the A strand and the N-terminus of the B strand were fairly well conserved and allowed us to model the relative location of PNGSs adjacent to these landmarks. None of the crystal structures described to date possessed asparagine at position 149, common among CRF01_AE viruses. With these caveats, our studies suggest that N136 and N149 occur on opposite ends of the hypervariable loop that connects the A and B strands (Fig. 5A). We also found that PNGSs at N136 and 149 appear to occur in close spatial proximity to the PNGSs at positions 301 and 332 at the stem of the V3 domain (Fig. 5B-5D). As can be seen in Fig. 5C-5D, the hypervariable connecting peptide between the A and B strands (cyan) appears to be located on the surface of the V1/V2 structure above the V3 domain (green) and fills the space between the N136 and N149 PNGSs (red, Fig. 5C-5D). According to our model, insertions of amino acids or PNGSs between these two sites would be predicted to sterically hinder the binding of bNAbs such as PGT121, PGT122, and PGT128 that bridge the glycans at N301 and N332 in the V3 domain and the N136 glycan in the V1/V2 domain.

This model appears to explain the differences in neutralization sensitivity observed between viruses from subjects 113035, 107747, and 142902 with respect to sensitivity to neutralization by the PGT128 bNAb. Thus the N149 PNGS conferred resistance to neutralization of the 113035 virus by PGT128 and by polyclonal antibodies from the T500208 and Z23 plasma. Deletion of the N149 resulted in a 16- to 53-fold increase in sensitivity to neutralization by PGT128. Paradoxically, 107747 and 142902 viruses were both sensitive to PGT128, yet possessed the N149 glycosylation site. Comparison of the sequences in the V1/V2 and V3 domains (Fig. 6) showed that all of the viruses from subject 113035 lacked the N301 PNGS in the V3 stem, whereas the viruses from 107747 and 142902 possessed the N301 PNGS thought to be important for PGT128 binding [22]. Our data suggest that the added stability provided by contact with the N301 glycan in the case of the 107747 and 142902 viruses overcomes the inhibitory effect of the N149 PNGS on the binding of PGT128-like neutralizing antibodies.

## Discussion

In these studies, we used genetic analysis of closely related variants in HIV-1 quasi-species to identify naturally occurring polymorphisms that confer resistance to neutralization in clade CRF01_AE viruses. While the determinants of neutralization sensitivity and resistance have been studied extensively in viruses from other clades, little information is available regarding CRF01_AE viruses. Understanding the determinants of neutralization sensitivity and resistance of clade CRF01_AE viruses is particularly important for HIV vaccine development, since two of the five large scale vaccine efficacy studies described to date (VAX003 and RV144) were carried out in Thailand [25,53], where viruses from this clade represented the majority of viruses in circulation. Several novel observations resulted from these studies. First, we identified V1 domain polymorphisms from three independent HIV infections that affected glycosylation sites and conferred neutralization sensitivity or resistance. Second, recent three dimensional structures of the HIV envelope glycoprotein have allowed us to localize these naturally occurring glycosylation site mutations to a specific structural element (i.e., the hypervariable connecting peptide between the A and B strands of V1/V2 domain). Third, neutralization resistance resulting from glycosylation at these sites was observed with plasma from a clade B infected subject (Z23) as well as CRF01_AE infected subjects, suggesting that viruses from both clades elicit neutralizing antibodies of a similar specificity. Finally, our studies suggest that the efficacy of HIV vaccines designed to provide protection against clade CRF01_AE might be expanded by the use of immunogens that lack the N136 and N149 N-linked glycosylation sites.

It has long been known that sequences in the V1/V2 domain are a major determinant of neutralization sensitivity and resistance [54,55]. For many years, antibodies to the V1/V2 domain were considered to be too strain specific and of little use in vaccines designed to elicit broad protective immunity. Later it was reported that the V1/V2 domain is important for conformational masking and serves to shield important parts of gp120 (e.g. V3 domain and CD4 binding site) from antibody binding [55–58]. Indeed, it was proposed that conformational masking by the V1/V2 domain inhibited the formation of neutralizing antibodies and that envelope proteins with deleted V2 domains might represent improved vaccine immunogens [56,59]. While deletion of the V2 domain enhanced immunogenicity and strain-specific neutralizing antibodies, it did not enhance the formation of bNAbs. In 2009, it was discovered that a major class of bNAbs in plasma from HIV-infected humans, the PG9 family, was directed to the V1/V2 domain and targeted GDEs involving PNGS at positions N156 and N160 [20,23]. Subsequently, it was reported that the PGT128 family of bNAbs depended on contacts with glycans at N136 in the V1 domain and at N301 and N332 in the stem of the V3 domain [18,21,24]. This represented a major advance in understanding the specificity of bNAbs and suggested that previous gp120 vaccines such as the AIDSVAX B/B and AIDSVAX B/E vaccines [60,61] used in the VAX003, VAX004, and RV144 trials [25,53,62] might be improved by incorporation of specific glycan structures required for the binding of bN-MAbs such as PG9, PGT121, and PGT128 [63,64].

In previous studies [13–15] we used swarm analysis to identify eight polymorphisms in clade B viruses, including three mutations in the V2 domain, three in gp41, and two in the CD4 binding site that conferred resistance to neutralization by bNAbs. In the present studies, we identified three mutations conferring neutralization resistance from three independent infections that all mapped to glycans in the V1 domain. This result raised the possibility that CRF01_AE viruses may have evolved a different strategy for immune escape than clade B viruses. This is consistent with the observation that CRF01_AE viruses typically lack the N332 glycosylation site required for binding by PGT121-like antibodies, and that this glycan contact cannot be replaced by glycans at 334 as is the case with viruses from other clades [22]. However, additional data will be required to test this hypothesis. In Fig. 5A, we have threaded the three V2 mutations that alter neutralization susceptibility in clade B viruses, and the two glycosylation site mutations identified in this study, onto the recent structure of trimeric gp140 [20]. Previously, we reported mutations that occurred at position 167 in the connecting peptide between the B and C strands, at position 179 in the connecting peptide between the C and D strands, and at a glycosylation site at position 197 at the end of the D strand [15]. In this study, we found that the sequences adjacent to the connecting peptide between the A and B strands similarly confer neutralization sensitivity and resistance. Thus the sequences at the ends and exposed turns of all four strands in the V1/V2 domain β-sheet structure all appear to modulate neutralization sensitivity and resistance.

The overall pattern of neutralization sensitivity and resistance that we observed at position 136 in the V1/V2 domain appears similar to the pattern of evolution of neutralization sensitivity and resistance described by Moore et al. [65] for antibodies targeting the V3 stem of clade C viruses. In that study, narrowly specific neutralizing antibodies that recognized the V3 stem were detected early after infection. Subsequently the virus evolved an N-linked glycosylation site at N332 that allowed the virus to escape neutralization by antibodies with narrow specificity. Later, however, the glycans at position N332 themselves became the target of glycan-dependent neutralizing antibodies that had expanded breadth of neutralization compared to the earlier antibodies targeting the non-glycosylated epitope. In our study, we detected antibodies in the plasma of three different individuals that were effective in neutralizing viruses from strains that lacked the N136 PNGS in the V1/V2 domain. The evolution of a glycosylation site at N136 resulted in variants resistant to these antibodies. We suspect that longitudinal analysis of antibodies in individuals that evolved the N136 glycosylation site would show the evolution of PGT121 and PGT122-like neutralizing antibodies targeting a GDE at N136 in a manner completely analogous to the evolution of the bNAbs targeting N332 described by Moore et al. [65]. However, the specimens required for longitudinal studies of this type are not available.

In conclusion, we have identified three independent mutations in the V1/V2 domain of CRF01_AE viruses, resulting in the creation of glycosylation sites (N136 and N149) that conferred resistance to neutralizing antibodies. Multiple studies have reported that transmitted/founder viruses have shorter V1/V2 domains with fewer PNGSs than viruses from chronic infections [66–68]. Comparative sequence analysis has shown that most of the variation in length and number of PNGSs between sequential isolates is due to insertions and mutations that create PNGSs in the hypervariable connecting peptides between the A and B strands and the C and D strands of the V1/V2 domain. It is notable that the vaccine immunogens (A244-rgp120 and vCP1521) used in the RV144 trial [53] both possessed V1/V2 sequences typical of chronic virus infections, with glycosylation sites at N136 and N149. Our data suggest that envelope glycoproteins that lack the N136 and N149 PNGSs in the V1/V2 domain may be more effective in eliciting neutralizing antibodies than the immunogens used in the RV144 trial.

## Supporting Information

S1 Table Neutralization sensitivity of pseudovirus constructed with envelopes from subject 107747. (PDF)

S2 Table Neutralization sensitivity of pseudovirus constructed with envelopes from subject 113035. (PDF)

S3 Table Neutralization sensitivity of pseudovirus constructed with envelopes from subject 142902. (PDF)
