# Supplementary material for: Glycans Flanking the Hypervariable Connecting Peptide between the A and B Strands of the V1/V2 Domain of HIV-1 gp120 Confer Resistance to Antibodies That Neutralize CRF01_AE Viruses
Source: PLoS One. 2015 Mar 20;10(3):e0119608. doi: 10.1371/journal.pone.0119608 (PMC4368187; doi:10.1371/journal.pone.0119608)
Supplement: S2 Table — (PDF) [file pone.0119608.s002.pdf]

**Supplemental Table S2. Neutralization sensitivity of pseudovirus constructed with envelopes from subject 113035**				
	**Neutralization titer (IC_{50}) obtained with HIV+ serum**			
**Clone / Serum**	**T500105**	**T500107**	**T500208**	**Z23**
**007 wtS**	<40	**616**	**555**	**399**
**012**	<40	**758**	**110**	**247**
**020**	**50**	**824**	**123**	**386**
**033**	<40	**722**	**253**	**284**
**036**	<40	**623**	**67**	**367**
**045 wtR**	<40	**1576**	**70**	**264**
**051**	<40	**587**	**59**	**276**
**055**	<40	**1355**	**162**	**306**
**070**	<40	**1281**	**81**	**246**
**081**	**133**	**729**	**103**	**336**
**JRCSF**	<40	**2611**	<40	**335**
**NL43**	**934**	**74**	**109**	**4852**
**aMLV**	<40	<40	<40	<100

The neutralizing antibody titer (IC_{50}) is defined as the reciprocal of the plasma dilution that produces a 50% inhibition in target cell infection. Values in bold represent significant neutralization titers that are at least three times greater than those observed against the negative control (aMLV). The Envs designated wildtype resistant (wtR) and wildtype sensitive (wtS) are indicated. Envs for both the wtR and wtS isolates were from CCR5-dependent viruses as determined by the Trofile® assay (Monogram Biosciences).
